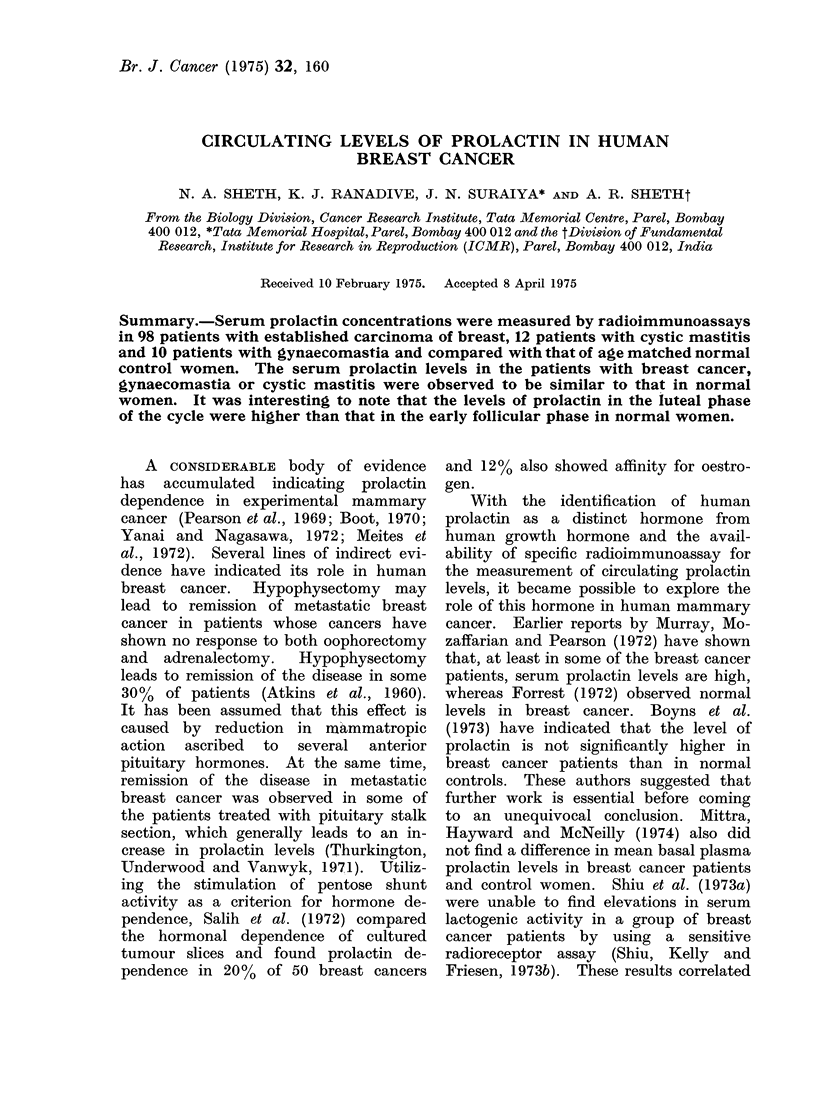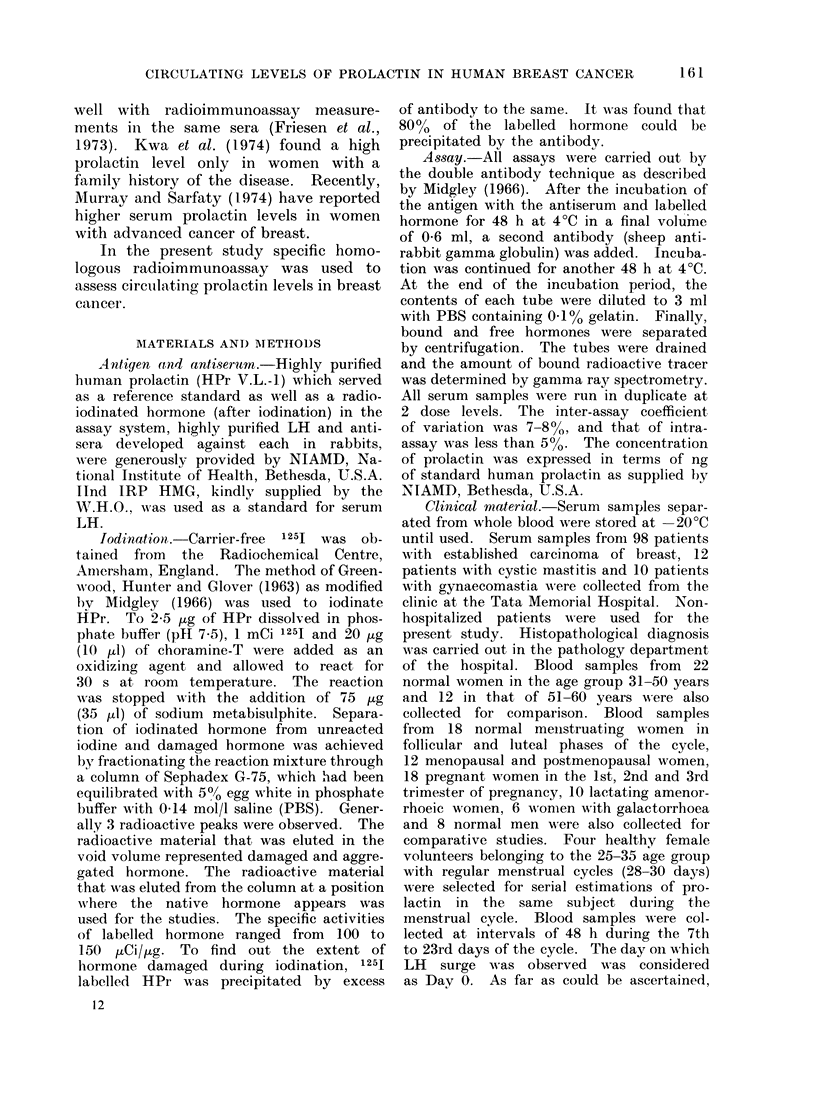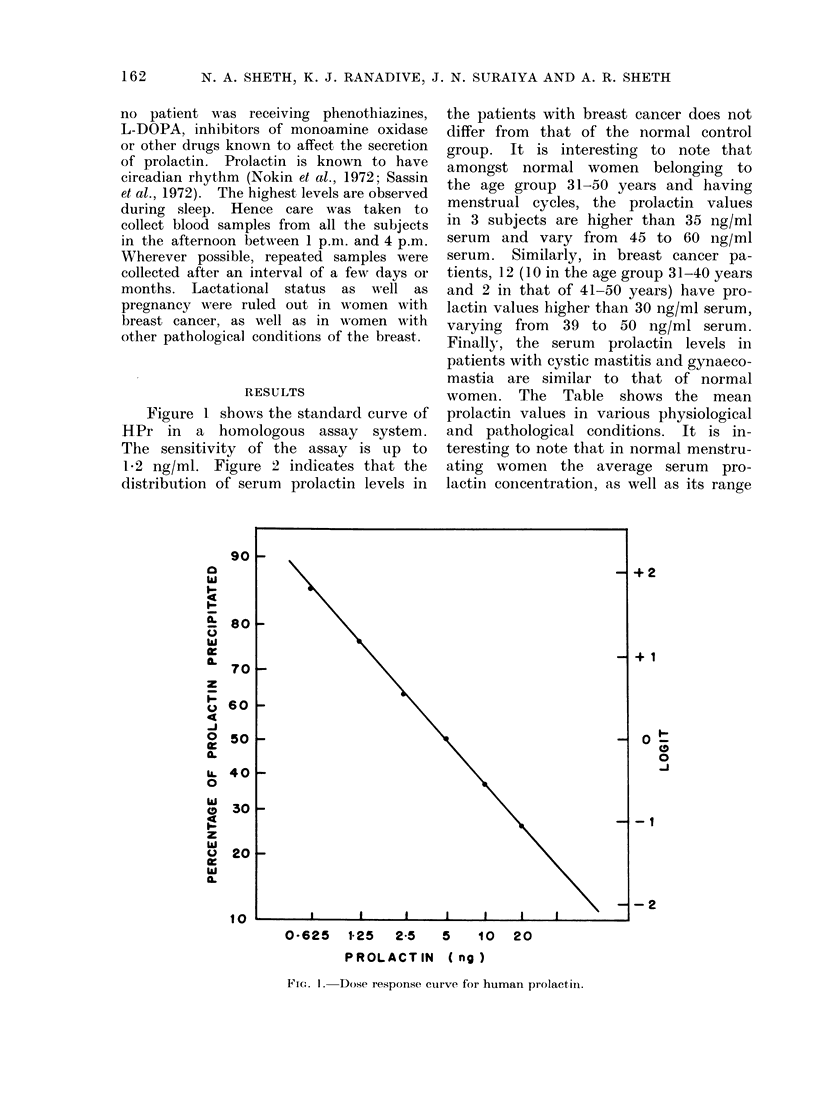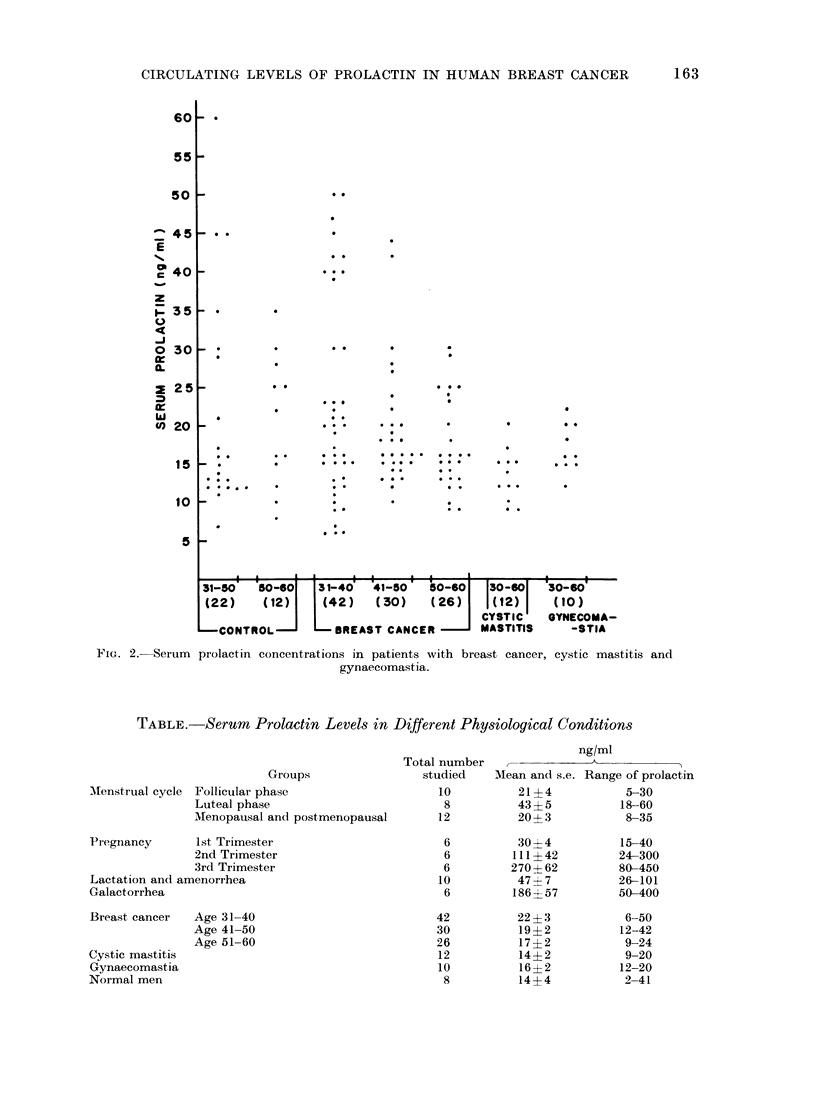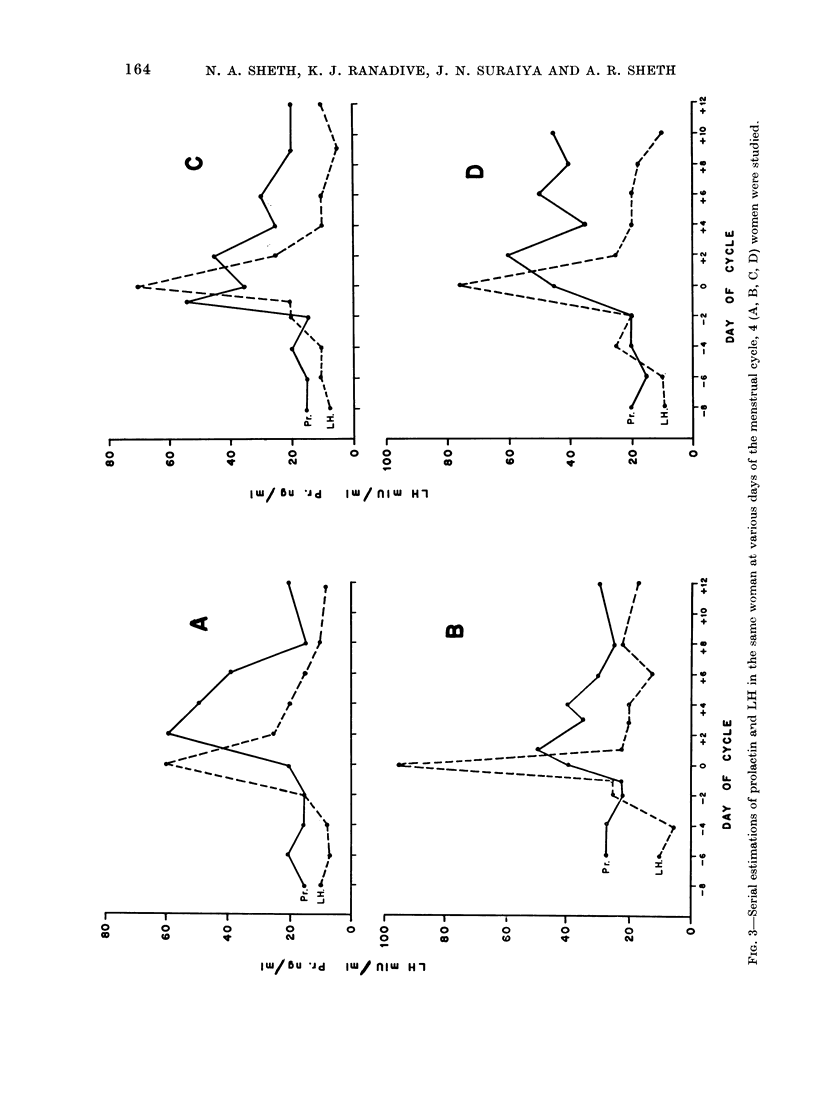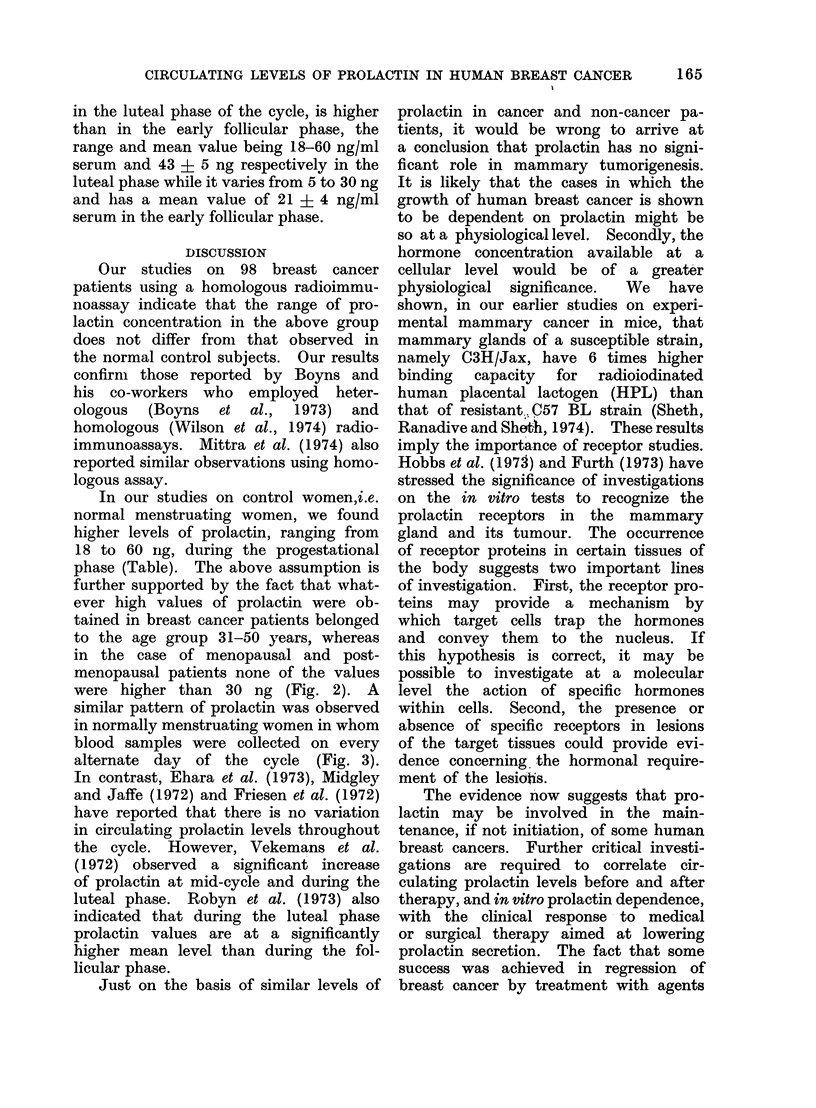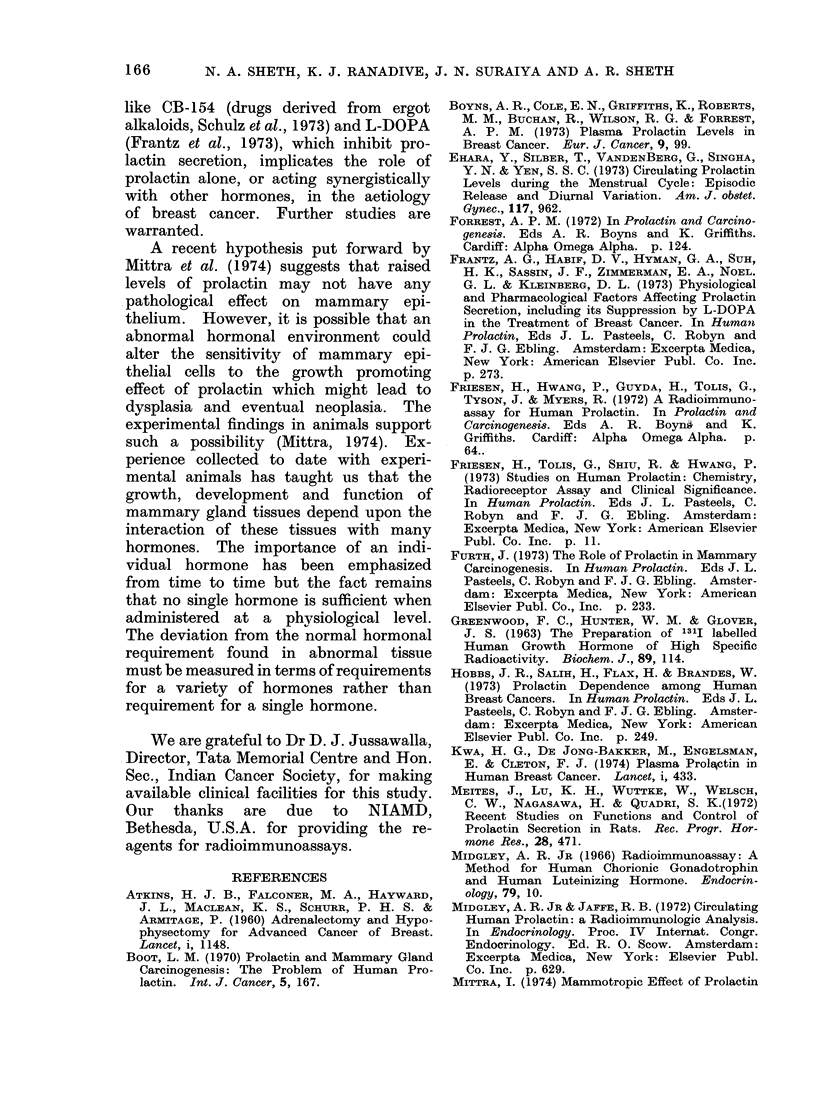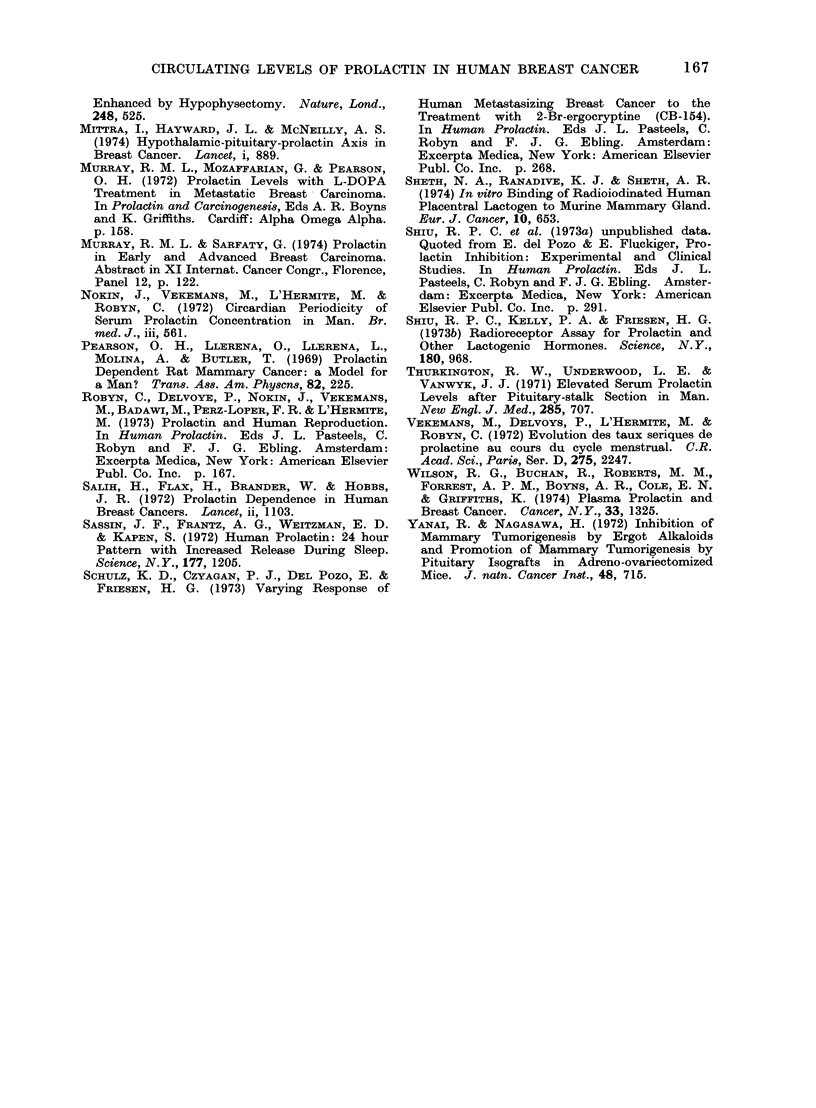# Circulating levels of prolactin in human breast cancer.

**DOI:** 10.1038/bjc.1975.145

**Published:** 1975-08

**Authors:** N. A. Sheth, K. J. Ranadive, J. N. Suraiya, A. R. Sheth

## Abstract

Serum prolactin concentrations were measured by radioimmunoassays in 98 patients with established carcinoma of breast, 12 patients with cystic mastitis and 10 patients with gynaecomastia and compared with that of age matched normal control women. The serum prolactin levels in the patients with breast cancer, gynaecomastia or cystic mastitis were observed to be similar to that in normal women. It was interesting to note that the levels of prolactin in the luteal phase of the cycle were higher than that in the early follicular phase in normal women.


*Br. J. Cancer* (1975) **32**, 160

### CIRCULATING LEVELS OF PROLACTIN IN HUMAN BREAST CANCER

N. A. SHETH, K. J. RANADIVE, J. N. SURAIYA* AND A. R. SHETH†

*From the Biology Division, Cancer Research Institute, Tata Memorial Centre, Parel, Bombay* 400 012, **Tata Memorial Hospital, Parel, Bombay* 400 012 *and the* †*Division of Fundamental Research, Institute for Research in Reproduction (ICMR), Parel, Bombay* 400 012, *India*

Received 10 February 1975. Accepted 8 April 1975

**Summary.—Serum prolactin concentrations were measured by radioimmunoassays in 98 patients with established carcinoma of breast, 12 patients with cystic mastitis and 10 patients with gynaecomastia and compared with that of age matched normal control women. The serum prolactin levels in the patients with breast cancer, gynaecomastia or cystic mastitis were observed to be similar to that in normal women. It was interesting to note that the levels of prolactin in the luteal phase of the cycle were higher than that in the early follicular phase in normal women.**

A CONSIDERABLE body of evidence has accumulated indicating prolactin dependence in experimental mammary cancer (Pearson *et al.*, 1969; Boot, 1970; Yanai and Nagasawa, 1972; Meites *et al.*, 1972). Several lines of indirect evidence have indicated its role in human breast cancer. Hypophysectomy may lead to remission of metastatic breast cancer in patients whose cancers have shown no response to both oophorectomy and adrenalectomy. Hypophysectomy leads to remission of the disease in some 30% of patients (Atkins *et al.*, 1960). It has been assumed that this effect is caused by reduction in mammatropic action ascribed to several anterior pituitary hormones. At the same time, remission of the disease in metastatic breast cancer was observed in some of the patients treated with pituitary stalk section, which generally leads to an increase in prolactin levels (Thurkington, Underwood and Vanwyk, 1971). Utilizing the stimulation of pentose shunt activity as a criterion for hormone dependence, Salih *et al.* (1972) compared the hormonal dependence of cultured tumour slices and found prolactin dependence in 20% of 50 breast cancers and 12% also showed affinity for oestrogen.

With the identification of human prolactin as a distinct hormone from human growth hormone and the availability of specific radioimmunoassay for the measurement of circulating prolactin levels, it became possible to explore the role of this hormone in human mammary cancer. Earlier reports by Murray, Mozaffarian and Pearson (1972) have shown that, at least in some of the breast cancer patients, serum prolactin levels are high, whereas Forrest (1972) observed normal levels in breast cancer. Boyns *et al.* (1973) have indicated that the level of prolactin is not significantly higher in breast cancer patients than in normal controls. These authors suggested that further work is essential before coming to an unequivocal conclusion. Mittra, Hayward and McNeilly (1974) also did not find a difference in mean basal plasma prolactin levels in breast cancer patients and control women. Shiu *et al.* (1973*a*) were unable to find elevations in serum lactogenic activity in a group of breast cancer patients by using a sensitive radioreceptor assay (Shiu, Kelly and Friesen, 1973*b*). These results correlated well with radioimmunoassay measurements in the same sera (Friesen *et al.*, 1973). Kwa *et al.* (1974) found a high prolactin level only in women with a family history of the disease. Recently, Murray and Sarfaty (1974) have reported higher serum prolactin levels in women with advanced cancer of breast.

In the present study specific homologous radioimmunoassay was used to assess circulating prolactin levels in breast cancer.

### MATERIALS AND METHODS

*Antigen and antiserum.*—Highly purified human prolactin (HPr V.L.-1) which served as a reference standard as well as a radioiodinated hormone (after iodination) in the assay system, highly purified LH and antisera developed against each in rabbits, were generously provided by NIAMD, National Institute of Health, Bethesda, U.S.A. IInd IRP HMG, kindly supplied by the W.H.O., was used as a standard for serum LH.

*Iodination.*—Carrier-free $^{125}I$ was obtained from the Radiochemical Centre, Amersham, England. The method of Greenwood, Hunter and Glover (1963) as modified by Midgley (1966) was used to iodinate HPr. To 2·5 $\mu$g of HPr dissolved in phosphate buffer (pH 7·5), 1 mCi $^{125}I$ and 20 $\mu$g (10 $\mu$l) of choramine-T were added as an oxidizing agent and allowed to react for 30 s at room temperature. The reaction was stopped with the addition of 75 $\mu$g (35 $\mu$l) of sodium metabisulphite. Separation of iodinated hormone from unreacted iodine and damaged hormone was achieved by fractionating the reaction mixture through a column of Sephadex G-75, which had been equilibrated with 5% egg white in phosphate buffer with 0·14 mol/l saline (PBS). Generally 3 radioactive peaks were observed. The radioactive material that was eluted in the void volume represented damaged and aggregated hormone. The radioactive material that was eluted from the column at a position where the native hormone appears was used for the studies. The specific activities of labelled hormone ranged from 100 to 150 $\mu$Ci/$\mu$g. To find out the extent of hormone damaged during iodination, $^{125}I$ labelled HPr was precipitated by excess of antibody to the same. It was found that 80% of the labelled hormone could be precipitated by the antibody.

*Assay.*—All assays were carried out by the double antibody technique as described by Midgley (1966). After the incubation of the antigen with the antiserum and labelled hormone for 48 h at 4°C in a final volume of 0·6 ml, a second antibody (sheep anti-rabbit gamma globulin) was added. Incubation was continued for another 48 h at 4°C. At the end of the incubation period, the contents of each tube were diluted to 3 ml with PBS containing 0·1% gelatin. Finally, bound and free hormones were separated by centrifugation. The tubes were drained and the amount of bound radioactive tracer was determined by gamma ray spectrometry. All serum samples were run in duplicate at 2 dose levels. The inter-assay coefficient of variation was 7–8%, and that of intra-assay was less than 5%. The concentration of prolactin was expressed in terms of ng of standard human prolactin as supplied by NIAMD, Bethesda, U.S.A.

*Clinical material.*—Serum samples separated from whole blood were stored at −20°C until used. Serum samples from 98 patients with established carcinoma of breast, 12 patients with cystic mastitis and 10 patients with gynaecomastia were collected from the clinic at the Tata Memorial Hospital. Non-hospitalized patients were used for the present study. Histopathological diagnosis was carried out in the pathology department of the hospital. Blood samples from 22 normal women in the age group 31–50 years and 12 in that of 51–60 years were also collected for comparison. Blood samples from 18 normal menstruating women in follicular and luteal phases of the cycle, 12 menopausal and postmenopausal women, 18 pregnant women in the 1st, 2nd and 3rd trimester of pregnancy, 10 lactating amenorrhoeic women, 6 women with galactorrhoea and 8 normal men were also collected for comparative studies. Four healthy female volunteers belonging to the 25–35 age group with regular menstrual cycles (28–30 days) were selected for serial estimations of prolactin in the same subject during the menstrual cycle. Blood samples were collected at intervals of 48 h during the 7th to 23rd days of the cycle. The day on which LH surge was observed was considered as Day 0. As far as could be ascertained, no patient was receiving phenothiazines, L-DOPA, inhibitors of monoamine oxidase or other drugs known to affect the secretion of prolactin. Prolactin is known to have circadian rhythm (Nokin *et al.*, 1972; Sassin *et al.*, 1972). The highest levels are observed during sleep. Hence care was taken to collect blood samples from all the subjects in the afternoon between 1 p.m. and 4 p.m. Wherever possible, repeated samples were collected after an interval of a few days or months. Lactational status as well as pregnancy were ruled out in women with breast cancer, as well as in women with other pathological conditions of the breast.

### RESULTS

Figure 1 shows the standard curve of HPr in a homologous assay system. The sensitivity of the assay is up to 1·2 ng/ml. Figure 2 indicates that the distribution of serum prolactin levels in the patients with breast cancer does not differ from that of the normal control group. It is interesting to note that amongst normal women belonging to the age group 31–50 years and having menstrual cycles, the prolactin values in 3 subjects are higher than 35 ng/ml serum and vary from 45 to 60 ng/ml serum. Similarly, in breast cancer patients, 12 (10 in the age group 31–40 years and 2 in that of 41–50 years) have prolactin values higher than 30 ng/ml serum, varying from 39 to 50 ng/ml serum. Finally, the serum prolactin levels in patients with cystic mastitis and gynaecomastia are similar to that of normal women. The Table shows the mean prolactin values in various physiological and pathological conditions. It is interesting to note that in normal menstruating women the average serum prolactin concentration, as well as its range

FIG. 1.—Dose response curve for human prolactin.

FIG. 2.—Serum prolactin concentrations in patients with breast cancer, cystic mastitis and gynaecomastia.

TABLE.—*Serum Prolactin Levels in Different Physiological Conditions*

| | Groups | Total number studied | ng/ml Mean and s.e. | ng/ml Range of prolactin |
|---|---|---|---|---|
| Menstrual cycle | Follicular phase | 10 | 21±4 | 5–30 |
| | Luteal phase | 8 | 43±5 | 18–60 |
| | Menopausal and postmenopausal | 12 | 20±3 | 8–35 |
| Pregnancy | 1st Trimester | 6 | 30±4 | 15–40 |
| | 2nd Trimester | 6 | 111±42 | 24–300 |
| | 3rd Trimester | 6 | 270±62 | 80–450 |
| Lactation and amenorrhea | | 10 | 47±7 | 26–101 |
| Galactorrhea | | 6 | 186±57 | 50–400 |
| Breast cancer | Age 31–40 | 42 | 22±3 | 6–50 |
| | Age 41–50 | 30 | 19±2 | 12–42 |
| | Age 51–60 | 26 | 17±2 | 9–24 |
| Cystic mastitis | | 12 | 14±2 | 9–20 |
| Gynaecomastia | | 10 | 16±2 | 12–20 |
| Normal men | | 8 | 14±4 | 2–41 |

FIG. 3—Serial estimations of prolactin and LH in the same woman at various days of the menstrual cycle, 4 (A, B, C, D) women were studied.

in the luteal phase of the cycle, is higher than in the early follicular phase, the range and mean value being 18–60 ng/ml serum and 43 $\pm$ 5 ng respectively in the luteal phase while it varies from 5 to 30 ng and has a mean value of 21 $\pm$ 4 ng/ml serum in the early follicular phase.

### DISCUSSION

Our studies on 98 breast cancer patients using a homologous radioimmunoassay indicate that the range of prolactin concentration in the above group does not differ from that observed in the normal control subjects. Our results confirm those reported by Boyns and his co-workers who employed heterologous (Boyns *et al.*, 1973) and homologous (Wilson *et al.*, 1974) radioimmunoassays. Mittra *et al.* (1974) also reported similar observations using homologous assay.

In our studies on control women,*i.e.* normal menstruating women, we found higher levels of prolactin, ranging from 18 to 60 ng, during the progestational phase (Table). The above assumption is further supported by the fact that whatever high values of prolactin were obtained in breast cancer patients belonged to the age group 31–50 years, whereas in the case of menopausal and post-menopausal patients none of the values were higher than 30 ng (Fig. 2). A similar pattern of prolactin was observed in normally menstruating women in whom blood samples were collected on every alternate day of the cycle (Fig. 3). In contrast, Ehara *et al.* (1973), Midgley and Jaffe (1972) and Friesen *et al.* (1972) have reported that there is no variation in circulating prolactin levels throughout the cycle. However, Vekemans *et al.* (1972) observed a significant increase of prolactin at mid-cycle and during the luteal phase. Robyn *et al.* (1973) also indicated that during the luteal phase prolactin values are at a significantly higher mean level than during the follicular phase.

Just on the basis of similar levels of prolactin in cancer and non-cancer patients, it would be wrong to arrive at a conclusion that prolactin has no significant role in mammary tumorigenesis. It is likely that the cases in which the growth of human breast cancer is shown to be dependent on prolactin might be so at a physiological level. Secondly, the hormone concentration available at a cellular level would be of a greater physiological significance. We have shown, in our earlier studies on experimental mammary cancer in mice, that mammary glands of a susceptible strain, namely C3H/Jax, have 6 times higher binding capacity for radioiodinated human placental lactogen (HPL) than that of resistant C57 BL strain (Sheth, Ranadive and Sheth, 1974). These results imply the importance of receptor studies. Hobbs *et al.* (1973) and Furth (1973) have stressed the significance of investigations on the *in vitro* tests to recognize the prolactin receptors in the mammary gland and its tumour. The occurrence of receptor proteins in certain tissues of the body suggests two important lines of investigation. First, the receptor proteins may provide a mechanism by which target cells trap the hormones and convey them to the nucleus. If this hypothesis is correct, it may be possible to investigate at a molecular level the action of specific hormones within cells. Second, the presence or absence of specific receptors in lesions of the target tissues could provide evidence concerning the hormonal requirement of the lesions.

The evidence now suggests that prolactin may be involved in the maintenance, if not initiation, of some human breast cancers. Further critical investigations are required to correlate circulating prolactin levels before and after therapy, and *in vitro* prolactin dependence, with the clinical response to medical or surgical therapy aimed at lowering prolactin secretion. The fact that some success was achieved in regression of breast cancer by treatment with agents like CB-154 (drugs derived from ergot alkaloids, Schulz *et al.*, 1973) and L-DOPA (Frantz *et al.*, 1973), which inhibit prolactin secretion, implicates the role of prolactin alone, or acting synergistically with other hormones, in the aetiology of breast cancer. Further studies are warranted.

A recent hypothesis put forward by Mittra *et al.* (1974) suggests that raised levels of prolactin may not have any pathological effect on mammary epithelium. However, it is possible that an abnormal hormonal environment could alter the sensitivity of mammary epithelial cells to the growth promoting effect of prolactin which might lead to dysplasia and eventual neoplasia. The experimental findings in animals support such a possibility (Mittra, 1974). Experience collected to date with experimental animals has taught us that the growth, development and function of mammary gland tissues depend upon the interaction of these tissues with many hormones. The importance of an individual hormone has been emphasized from time to time but the fact remains that no single hormone is sufficient when administered at a physiological level. The deviation from the normal hormonal requirement found in abnormal tissue must be measured in terms of requirements for a variety of hormones rather than requirement for a single hormone.

We are grateful to Dr D. J. Jussawalla, Director, Tata Memorial Centre and Hon. Sec., Indian Cancer Society, for making available clinical facilities for this study. Our thanks are due to NIAMD, Bethesda, U.S.A. for providing the reagents for radioimmunoassays.

REFERENCES

Atkins, H. J. B., Falconer, M. A., Hayward, J. L., Maclean, K. S., Schurr, P. H. S. & Armitage, P. (1960) Adrenalectomy and Hypophysectomy for Advanced Cancer of Breast. *Lancet*, i, 1148.

Boot, L. M. (1970) Prolactin and Mammary Gland Carcinogenesis: The Problem of Human Prolactin. *Int. J. Cancer*, **5**, 167.

Boyns, A. R., Cole, E. N., Griffiths, K., Roberts, M. M., Buchan, R., Wilson, R. G. & Forrest, A. P. M. (1973) Plasma Prolactin Levels in Breast Cancer. *Eur. J. Cancer*, **9**, 99.

Ehara, Y., Silber, T., VandenBerg, G., Singha, Y. N. & Yen, S. S. C. (1973) Circulating Prolactin Levels during the Menstrual Cycle: Episodic Release and Diurnal Variation. *Am. J. obstet. Gynec.*, **117**, 962.

Forrest, A. P. M. (1972) In *Prolactin and Carcinogenesis*. Eds A. R. Boyns and K. Griffiths. Cardiff: Alpha Omega Alpha. p. 124.

Frantz, A. G., Habif, D. V., Hyman, G. A., Suh, H. K., Sassin, J. F., Zimmerman, E. A., Noel. G. L. & Kleinberg, D. L. (1973) Physiological and Pharmacological Factors Affecting Prolactin Secretion, including its Suppression by L-DOPA in the Treatment of Breast Cancer. In *Human Prolactin*, Eds J. L. Pasteels, C. Robyn and F. J. G. Ebling. Amsterdam: Excerpta Medica, New York: American Elsevier Publ. Co. Inc. p. 273.

Friesen, H., Hwang, P., Guyda, H., Tolis, G., Tyson, J. & Myers, R. (1972) A Radioimmunoassay for Human Prolactin. In *Prolactin and Carcinogenesis*. Eds A. R. Boyns and K. Griffiths. Cardiff: Alpha Omega Alpha. p. 64..

Friesen, H., Tolis, G., Shiu, R. & Hwang, P. (1973) Studies on Human Prolactin: Chemistry, Radioreceptor Assay and Clinical Significance. In *Human Prolactin*. Eds J. L. Pasteels, C. Robyn and F. J. G. Ebling. Amsterdam: Excerpta Medica, New York: American Elsevier Publ. Co. Inc. p. 11.

Furth, J. (1973) The Role of Prolactin in Mammary Carcinogenesis. In *Human Prolactin*. Eds J. L. Pasteels, C. Robyn and F. J. G. Ebling. Amsterdam: Excerpta Medica, New York: American Elsevier Publ. Co., Inc. p. 233.

Greenwood, F. C., Hunter, W. M. & Glover, J. S. (1963) The Preparation of $^{131}$I labelled Human Growth Hormone of High Specific Radioactivity. *Biochem. J.*, **89**, 114.

Hobbs, J. R., Salih, H., Flax, H. & Brandes, W. (1973) Prolactin Dependence among Human Breast Cancers. In *Human Prolactin*. Eds J. L. Pasteels, C. Robyn and F. J. G. Ebling. Amsterdam: Excerpta Medica, New York: American Elsevier Publ. Co. Inc. p. 249.

Kwa, H. G., De Jong-Bakker, M., Engelsman, E. & Cleton, F. J. (1974) Plasma Prolactin in Human Breast Cancer. *Lancet*, i, 433.

Meites, J., Lu, K. H., Wuttke, W., Welsch, C. W., Nagasawa, H. & Quadri, S. K.(1972) Recent Studies on Functions and Control of Prolactin Secretion in Rats. *Rec. Progr. Hormone Res.*, **28**, 471.

Midgley, A. R. Jr (1966) Radioimmunoassay: A Method for Human Chorionic Gonadotrophin and Human Luteinizing Hormone. *Endocrinology*, **79**, 10.

Midgley, A. R. Jr & Jaffe, R. B. (1972) Circulating Human Prolactin: a Radioimmunologic Analysis. In *Endocrinology*. Proc. IV Internat. Congr. Endocrinology. Ed. R. O. Scow. Amsterdam: Excerpta Medica, New York: Elsevier Publ. Co. Inc. p. 629.

Mittra, I. (1974) Mammotropic Effect of Prolactin

Enhanced by Hypophysectomy. *Nature, Lond.*, **248**, 525.

MITTRA, I., HAYWARD, J. L. & MCNEILLY, A. S. (1974) Hypothalamic-pituitary-prolactin Axis in Breast Cancer. *Lancet*, i, 889.

MURRAY, R. M. L., MOZAFFARIAN, G. & PEARSON, O. H. (1972) Prolactin Levels with L-DOPA Treatment in Metastatic Breast Carcinoma. In *Prolactin and Carcinogenesis*, Eds A. R. Boyns and K. Griffiths. Cardiff: Alpha Omega Alpha. p. 158.

MURRAY, R. M. L. & SARFATY, G. (1974) Prolactin in Early and Advanced Breast Carcinoma. Abstract in XI Internat. Cancer Congr., Florence, Panel 12, p. 122.

NOKIN, J., VEKEMANS, M., L'HERMITE, M. & ROBYN, C. (1972) Circardian Periodicity of Serum Prolactin Concentration in Man. *Br. med. J.*, iii, 561.

PEARSON, O. H., LLERENA, O., LLERENA, L., MOLINA, A. & BUTLER, T. (1969) Prolactin Dependent Rat Mammary Cancer: a Model for a Man? *Trans. Ass. Am. Physcns*, **82**, 225.

ROBYN, C., DELVOYE, P., NOKIN, J., VEKEMANS, M., BADAWI, M., PERZ-LOPER, F. R. & L'HERMITE, M. (1973) Prolactin and Human Reproduction. In *Human Prolactin*. Eds J. L. Pasteels, C. Robyn and F. J. G. Ebling. Amsterdam: Excerpta Medica, New York: American Elsevier Publ. Co. Inc. p. 167.

SALIH, H., FLAX, H., BRANDER, W. & HOBBS, J. R. (1972) Prolactin Dependence in Human Breast Cancers. *Lancet*, ii, 1103.

SASSIN, J. F., FRANTZ, A. G., WEITZMAN, E. D. & KAPEN, S. (1972) Human Prolactin: 24 hour Pattern with Increased Release During Sleep. *Science, N.Y.*, **177**, 1205.

SCHULZ, K. D., CZYAGAN, P. J., DEL POZO, E. & FRIESEN, H. G. (1973) Varying Response of Human Metastasizing Breast Cancer to the Treatment with 2-Br-ergocryptine (CB-154). In *Human Prolactin*. Eds J. L. Pasteels, C. Robyn and F. J. G. Ebling. Amsterdam: Excerpta Medica, New York: American Elsevier Publ. Co. Inc. p. 268.

SHETH, N. A., RANADIVE, K. J. & SHETH, A. R. (1974) *In vitro* Binding of Radioiodinated Human Placentral Lactogen to Murine Mammary Gland. *Eur. J. Cancer*, **10**, 653.

SHIU, R. P. C. *et al.* (1973*a*) unpublished data. Quoted from E. del Pozo & E. Fluckiger, Prolactin Inhibition: Experimental and Clinical Studies. In *Human Prolactin*. Eds J. L. Pasteels, C. Robyn and F. J. G. Ebling. Amsterdam: Excerpta Medica, New York: American Elsevier Publ. Co. Inc. p. 291.

SHIU, R. P. C., KELLY, P. A. & FRIESEN, H. G. (1973*b*) Radioreceptor Assay for Prolactin and Other Lactogenic Hormones. *Science, N.Y.*, **180**, 968.

THURKINGTON, R. W., UNDERWOOD, L. E. & VANWYK, J. J. (1971) Elevated Serum Prolactin Levels after Pituitary-stalk Section in Man. *New Engl. J. Med.*, **285**, 707.

VEKEMANS, M., DELVOYS, P., L'HERMITE, M. & ROBYN, C. (1972) Evolution des taux seriques de prolactine au cours du cycle menstrual. *C.R. Acad. Sci., Paris*, Ser. D, **275**, 2247.

WILSON, R. G., BUCHAN, R., ROBERTS, M. M., FORREST, A. P. M., BOYNS, A. R., COLE, E. N. & GRIFFITHS, K. (1974) Plasma Prolactin and Breast Cancer. *Cancer, N.Y.*, **33**, 1325.

YANAI, R. & NAGASAWA, H. (1972) Inhibition of Mammary Tumorigenesis by Ergot Alkaloids and Promotion of Mammary Tumorigenesis by Pituitary Isografts in Adreno-ovariectomized Mice. *J. natn. Cancer Inst.*, **48**, 715.